# The Liganding of Glycolipid Transfer Protein Is Controlled by Glycolipid Acyl Structure

**DOI:** 10.1371/journal.pbio.0040362

**Published:** 2006-10-24

**Authors:** Lucy Malinina, Margarita L Malakhova, Alex T Kanack, Min Lu, Ruben Abagyan, Rhoderick E Brown, Dinshaw J Patel

**Affiliations:** 1 Structural Biology Program, Memorial Sloan-Kettering Cancer Center, New York, New York, United States of America; 2 Hormel Institute, University of Minnesota, Austin, Minnesota, United States of America; 3 Department of Biochemistry, Weill Medical College of Cornell University, New York, New York, United States of America; 4 Department of Molecular Biology, The Scripps Research Institute, La Jolla, California, United States of America; Princeton University, United States of America

## Abstract

Glycosphingolipids (GSLs) play major roles in cellular growth and development. Mammalian glycolipid transfer proteins (GLTPs) are potential regulators of cell processes mediated by GSLs and display a unique architecture among lipid binding/transfer proteins. The GLTP fold represents a novel membrane targeting/interaction domain among peripheral proteins. Here we report crystal structures of human GLTP bound to GSLs of diverse acyl chain length, unsaturation, and sugar composition. Structural comparisons show a highly conserved anchoring of galactosyl- and lactosyl-amide headgroups by the GLTP recognition center. By contrast, acyl chain chemical structure and occupancy of the hydrophobic tunnel dictate partitioning between sphingosine-in and newly-observed sphingosine-out ligand-binding modes. The structural insights, combined with computed interaction propensity distributions, suggest a concerted sequence of events mediated by GLTP conformational changes during GSL transfer to and/or from membranes, as well as during GSL presentation and/or transfer to other proteins.

## Abstract 2

Structures of glycosphingolipids bound to glycolipid transfer proteins reveal a sphingosine-in or -out binding depending on their acyl chain structure; these conformational changes suggest a mechanism for glycosphingolipid transfer.

## Introduction

Glycosphingolipids (GSLs) are ubiquitous components of eukaryotic plasma membranes and mediate numerous biological functions, from differentiation and proliferation to invasive adhesion, neurodegeneration, and apoptosis [1,2]. The importance of GSLs and their metabolites in processes such as tissue development is illustrated by the lethal effect of disruption of GSL biosynthesis during embryonic development [3]. Whereas the bulk of GSLs reside in the outer leaflet of the plasma membrane where their sugar headgroups project into the extracellular environment, increasing evidence suggests that GSLs also localize to membranes of intracellular organelles (e.g., nucleus and mitochondria) [4–6]. Vesicular trafficking plays a dominant role in distributing GSLs intracellularly after synthesis in the Golgi. However, a nonvesicular pathway also exists, possibly involving glycolipid transfer proteins (GLTPs) [7,8].

Based on their ability to selectively accelerate the intermembrane transfer of glycolipids, GLTPs were discovered in the cytosolic extracts of bovine spleen and porcine brain, and later in a wide variety of tissues [9–11]. The 23–24-kDa GLTPs display absolute specificity for glycolipids [12], are highly conserved among mammals [13], and include plant and fungal orthologs that have been implicated in programmed cell death responses [14,15]. Recently, we determined the first x-ray structure of human GLTP in both the GSL-free and lactosylceramide (LacCer)-bound forms [16]. In addition to displaying a novel architecture that defines GLTP as the founding member of a new protein superfamily [17,18] (see http://supfam.org/SUPERFAMILY/cgi-bin/scop.cgi?sunid=110004 and http://scop.mrc-lmb.cam.ac.uk/scop-1.69/data/scop.b.b.bja.b.b.A.html; accessed 12 September 2006) with a novel protein fold for membrane interaction and for lipid binding/transfer [19–27], the structure of the 18:1 LacCer-GLTP complex revealed the basis for GSL binding specificity by GLTP. The liganding site consists of a sugar headgroup recognition center that anchors the ceramide-linked sugar to the protein surface and a hydrophobic tunnel that accommodates the hydrocarbon chains of ceramide. Comparative structural analyses, including crystallographic B-factor distributions of apo-GLTP and the LacCer-GLTP complex, suggest that liganding of the glycolipid most likely occurs via an adaptive recognition process [16]. A cleft-like gating mechanism, involving conformational changes to two interhelical loops and one α helix, appears to facilitate entry and exit of the lipid chains in the membrane-associated state when the GSL headgroup is attached to the sugar headgroup recognition center.

An important feature of the GLTP hydrophobic tunnel is its ability to expand to accommodate the GSL ceramide moiety. In the case of the 18:1 LacCer-GLTP complex, the 18-carbon oleoyl and sphingosine chains of LacCer reside side by side within the hydrophobic channel [16]. However, naturally occurring mammalian glycolipids typically have acyl chains with lengths ranging from 16 to 26 carbons with occasional monounsaturation. To test the conformational properties and accommodation limits of the GLTP hydrophobic tunnel, we synthesized glycolipids (LacCer or galactosylceramide [GalCer]) containing short acyl chains (e.g., octanoyl and dodecanoyl), medium unsaturated acyl chains (e.g., linoleoyl), as well as long, physiologically relevant unsaturated acyl chains (e.g., nervonyl), and we structurally characterized their complexes with GLTP. To elucidate conformational changes caused by the sugar headgroup binding in the GLTP, we also structurally characterized the complex between GLTP and the model compound *n*-hexyl-β-d-glycoside, which, unlike GSLs, has a single, very short hydrocarbon chain lacking an amide linkage.

The present structural studies provide definitive evidence for a novel GSL-GLTP complexation mode characterized by a difference in the accommodation of the ceramide lipid chains but not the sugar headgroups. Chief among the differences is bending of the sphingosine chain, causing outward projection from the hydrophobic tunnel. Comparative analysis of all GSL-GLTP structures reveals that the hydrophobic tunnel consists of two functionally distinct compartments, enabling GLTP to accommodate GSL acyl chains of different lengths and conformational restrictions. Equally importantly, our findings suggest a concerted sequence of events during GSL liganding, in which the sphingosine chain is the last part of the glycolipid to enter GLTP and the first part to leave GLTP during interaction with membranes. Interaction propensity distribution computations indicate a potential GLTP interface for interactions with other proteins and/or membranes, which coincide with a region of the proposed GLTP gate mediating GSL binding and release.

## Results

### Nonpolar Nature of GLTP Hydrophobic Channel Promotes Occupancy by Nonpolar Lipids

Our original structural characterization of GLTP revealed two noteworthy features of the hydrophobic tunnel that encapsulates the GSL hydrocarbon chains [16]. First, the tunnel is highly nonpolar. No water molecules reside in the channel, which is lined by the side chains of nonpolar phenylalanine, leucine, isoleucine, and alanine residues, together with a few valine and proline residues. Second, a large portion of the tunnel is conformationally adaptable and expands during GSL acquisition to accommodate the hydrocarbon chains. Moreover, localized in the lower part of the hydrophobic tunnel of apo-GLTP (i.e., glycolipid-free GLTP) is an extraneous hydrocarbon molecule that contains at least six carbon atoms and is clearly observable in electron density maps (Figure S1). Consistent with this finding is a recent report of decanoic acid (ten-carbon chain) within the hydrophobic tunnels of two glycolipid-free bovine GLTP structures [28]. It is not clear if the extraneous hydrocarbon is acquired during heterologous expression in Escherichia coli or subsequent crystallization*.* However, the situation is not unusual for GSL binding proteins [22] and also has been frequently observed among other lipid binding/transfer proteins [20].

The extraneous hydrocarbon is displaced from the hydrophobic tunnel when human GLTP forms a complex with 18:1 LacCer (Figure S2). A similar displacement of decanoic acid from the hydrophobic tunnel also seems to occur when bovine GLTP complexes with ganglioside GM3 [28].

### Structure of the 24:1 GalCer-GLTP Complex Containing Long Acyl Chain GSL

**Figure 1:**
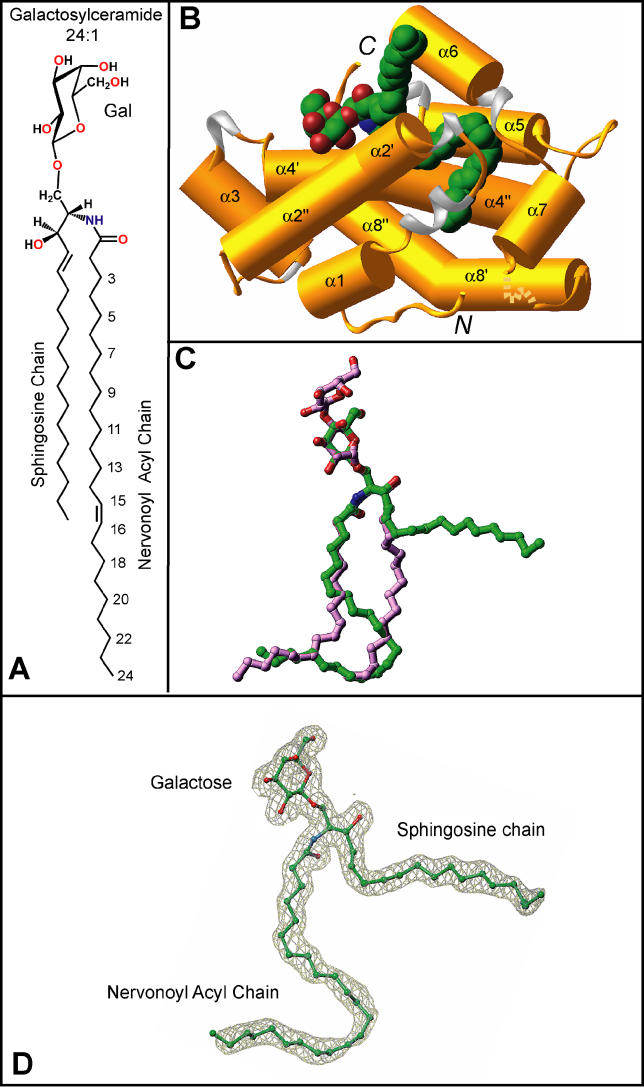
Structure of the 24:1 GalCer-GLTP Complex (A) Chemical formula of 24:1 galactosylceramide. (B) Crystal structure of the 24:1 GalCer-GLTP complex. α helices (colored gold) are shown in a cylinder representation, 3_10_ helices (colored silver) and loop regions (colored gold) are in ribbon representations, and bound GalCer is in a space-filling representation. The glycolipid atoms are colored green, red, and blue for carbon, oxygen, and nitrogen atoms, respectively. (C) Superposition of stick representations of 24:1 GalCer (carbon atoms colored in green) and 18:1 LacCer (carbon atoms colored in lavender) derived from their sphingosine-out and sphingosine-in GLTP complexes, respectively. (D) The 24:1 GalCer ligand structure inside the simulated annealed omit 2*F*_o_-*F*_c_ map contoured at 1σ level. The color code for the glycolipid atoms is the same as in (B).

To determine how GSLs with long and physiologically relevant acyl chains are accommodated within the hydrophobic channel, GLTP was cocrystallized with GalCer containing a nervonoyl acyl chain (24-carbon chain with a *cis*-15,16 double bond, 24:1^Δ15cis^) (Figure 1A) under conditions described previously [16] for complexation of GLTP with LacCer containing an oleoyl acyl chain (18:1^Δ9cis^) (Figure S2A). The crystal structure of 24:1 GalCer bound to GLTP (Figure 1) was refined to an *R* factor (measure of goodness of fit) of 18.0% at 1.85 Å resolution (Table 1) (see Materials and Methods), enabling construction of an uninterrupted and clear electron density map for the sugar-amide headgroup and both ceramide lipid chains (Figure 1D). The overall α-helical topology of the 24:1 GalCer-GLTP complex (Figure 1B) and the conformational features of its glycolipid-binding region are very similar to that reported previously for our structure of the 18:1 LacCer-GLTP complex (Figure S2B) [16] and recently confirmed in bovine His_6_-tagged GLTP [28]. Two long α helices (helices 8 and 4) intertwine to form a super-helix, with both being noticeably bent (Figure 1B). One of these helices (helix 4) interacts directly with 24:1 GalCer, with one segment supporting the polar headgroup and the remainder supporting the nonpolar lipid chains.

**Table 1:**
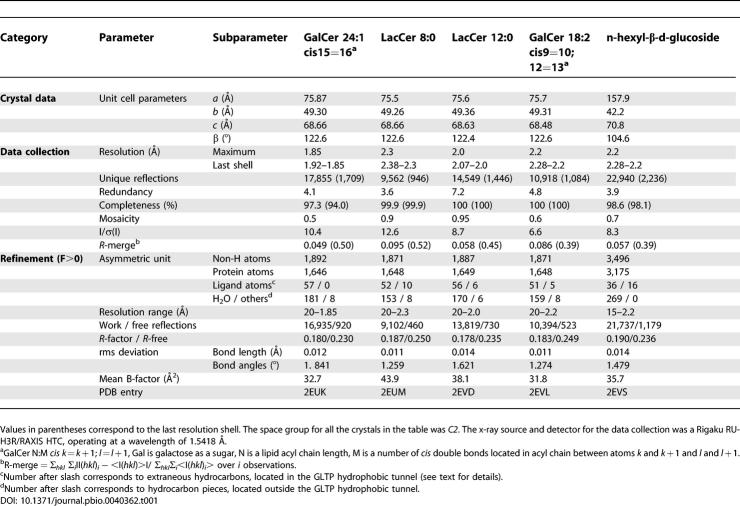
X-Ray Data Collection and Refinement Statistics

### Galactose Versus Lactose in the GLTP Recognition Center

**Figure 2:**
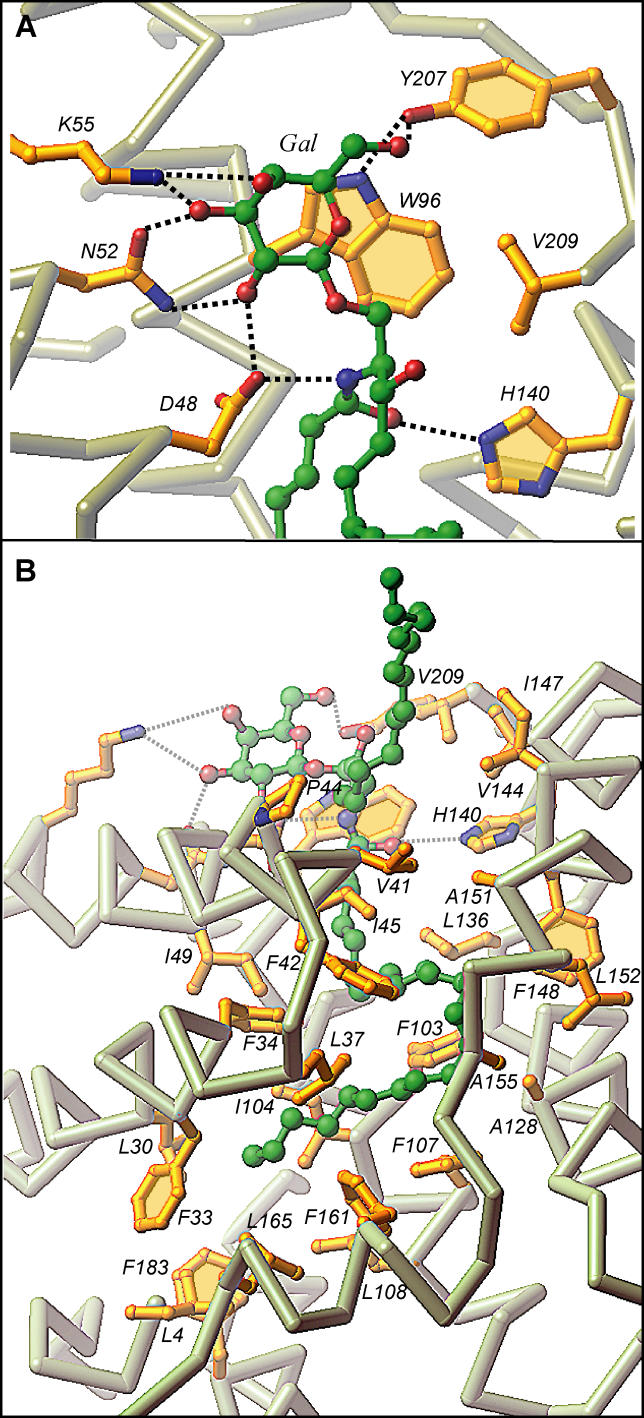
GSL-GLTP Interactions in the 24:1 GalCer-GLTP Complex (A) 24:1 GalCer headgroup (sugar and amide) interactions with GLTP recognition center residues. Hydrogen bonds are shown by dashed lines. The bound GSL atoms are colored by green, red, and blue for carbon, oxygen, and nitrogen atoms, respectively. The GLTP C_α_ backbone is colored light gray, the side chains are shown in gold, and oxygen and nitrogen are red and blue, respectively. (B) 24:1 GalCer lipid interactions with the GLTP channel residues. The longer acyl chain is directed into the channel while the shorter sphingosine chain is directed outwards.

In the 24:1 GalCer-GLTP complex, the galactose sugar headgroup is anchored within the GLTP recognition center, which is located on the protein surface, through a network of hydrogen bonding and hydrophobic interactions (Figure 2A). The hydrogen bonding involves D48, N52, and K55 of α helix 2 and Y207 near the C terminus. The stacking of W96 (α helix 4) against the B face of the galactose ring (Figure 2A) is similar to that with the glucose ring in 18:1 LacCer complex [16]. The only difference in the liganding between the single sugar headgroup of 24:1 GalCer and the two-sugar headgroup of 18:1 LacCer involves K55, which hydrogen bonds with the OH3 and OH4 hydroxyls of the initial sugar ring attached to the ceramide in 24:1 GalCer (Figure 2A), rather than hydrogen bonding with the OH3 hydroxyl of the second sugar residue in the lactose of the 18:1 LacCer [16].

Orientation of the ceramide amide group of 24:1 GalCer is controlled by a pair of hydrogen bonds involving D48 and H140, with alignment of the initial segment of the sphingoid base being facilitated by van der Waals contacts with V209 (Figure 2A). A similar situation occurs in the 18:1 LacCer-GLTP complex [16]. The interactions are important because they result in a conserved and oriented entry of the GSL ceramide lipid tails into the hydrophobic channel of GLTP.

### Accommodation of a Long Acyl Chain GSL by GLTP

Accommodation of the ceramide lipid tails within the hydrophobic channel in the 24:1 GalCer-GLTP complex (Figure 1B) differs strikingly from that observed in the 18:1 LacCer-GLTP complex (Figure S2B) [16]. Only the long nervonoyl acyl chain is inserted into the hydrophobic tunnel, which is lined by the nonpolar side chains of multiple phenylalanine, leucine, isoleucine, and alanine residues in the 24:1 GalCer-GLTP complex (longer green-colored lipid chain in Figures 1B and 2B). The sphingosine chain of 24:1 GalCer bends sharply at carbon 6, and projects nearly orthogonally away from the cleft-like gate of the tunnel (shorter green-colored lipid chain in Figures 1B and 2B). Such an arrangement contrasts with that of the 18:1 LacCer-GLTP complex, in which substantial portions of both the oleoyl acyl and sphingosine chains are buried within the hydrophobic tunnel (Figure S2B) in parallel fashion (Figure S2C). Furthermore, to accommodate the six additional methylenes of the 24:1 acyl chain in GalCer, the nervonoyl chain curves in a serpentine fashion (Figure 1B) within the tunnel (longer green-colored lipid chain in Figure 1C), occupying the region filled by the sphingosine chain in the 18:1 LacCer complex (shorter lavender colored lipid chain in Figure 1C), and interfering with entry of the sphingosine chain of 24:1 GalCer into the tunnel.

### Classification of GLTP and Its GSL-GLTP Complexes

**Figure 3:**
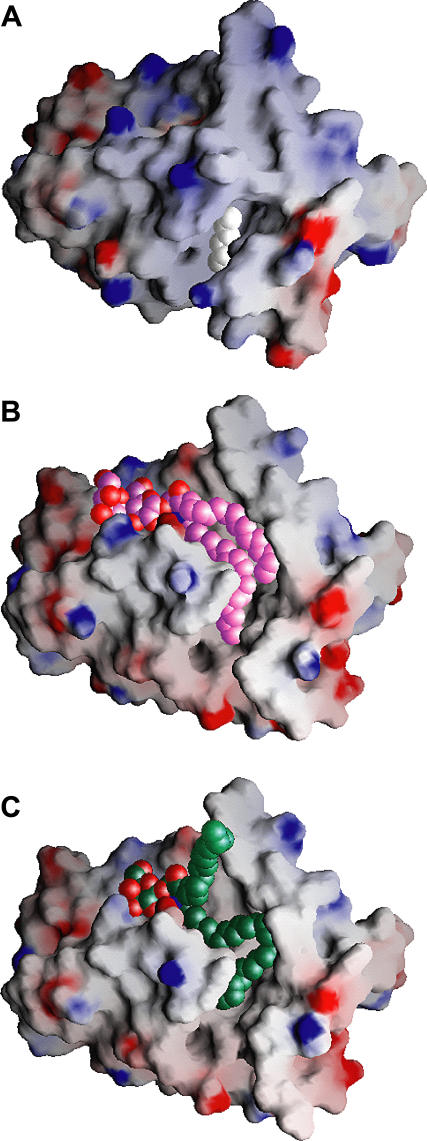
Gate-Removed Electrostatic Surface Views of the GLTP Hydrophobic Tunnel Accommodating GSLs and/or Extraneous Hydrocarbons (A) Structure of apo-GLTP. The GLTP is shown in an electrostatic surface representation (blue, positive; red, negative; gray, neutral), with gate residues 33 and 35 to 39 removed to make the tunnel visible in (A), and residues 33 and 35 to 45 removed in (B) and (C). The view for apo-GLTP shows the collapsed upper part of the tunnel and bound extraneous hydrocarbon positioned within the uncollapsed bottom of the tunnel. The carbon atoms of the extraneous hydrocarbon are shown in a white space-filling representation. (B) Structure of the 18:1 LacCer-GLTP complex exhibiting the sphingosine-in mode. The carbon atoms of the 18:1 LacCer are shown in a lavender space-filling representation. Both lipid chains optimally fit into the available space of the hydrophobic tunnel. (C) Structure of the 24:1 GalCer-GLTP complex exhibiting the sphingosine-out mode. The carbon atoms of the 24:1 GalCer are shown in a green space-filling representation. The long acyl chain, bent in a serpentine fashion, occupies the available space of the hydrophobic tunnel, resulting in an outward positioning of the sphingosine chain.

The structures of GLTP and its GSL complexes can be classified into three families. The hydrophobic tunnel is closed in apo-GLTP (cut-away view, Figure 3A) [16], whereas it is open and maximally expanded in the sphingosine-in conformation observed for 18:1 LacCer-GLTP complex (cut-away view, Figure 3B) [16] and the sphingosine-out conformation of the 24:1 GalCer-GLTP complex (cut-away view, Figure 3C).

**Figure 4:**
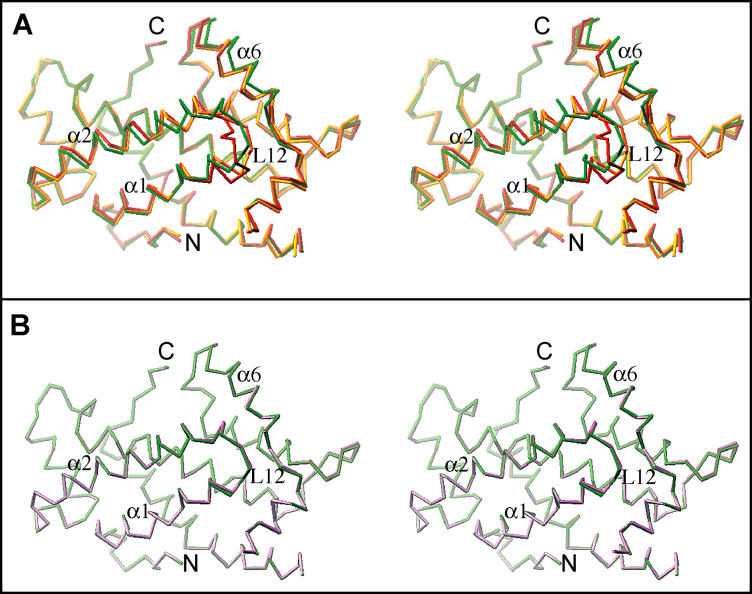
Stereo Superposition of the Conformational States of GLTP (A) The GLTP backbone is shown in red, gold, or green C_α_ representation for apo-GLTP and GLTP complexes with *n*-hexyl-β-d-glucoside and 24:1 GalCer, respectively. (B) The GLTP C_α_ backbone is shown in green and lavender for GLTP complexes with 24:1 GalCer and 18:1 LacCer, respectively.

GLTP undergoes a conformation change on proceeding from apo-GLTP (colored red, stereo view in Figure 4A) [16] to its 24:1 GalCer-GLTP complex (colored green, stereo view in Figure 4A). Despite the differences in the sphingosine-in and sphingosine-out alignments, the GLTP conformations are very similar in the 18:1 LacCer-GLTP complex (colored lavender, stereo view in Figure 4B) [16] and the newly identified 24:1 GalCer-GLTP complex (colored green, stereo view in Figure 4B).

### GLTP Complex with *n*-hexyl-β-d-glucoside That Lacks Ceramide Moiety

**Figure 5:**
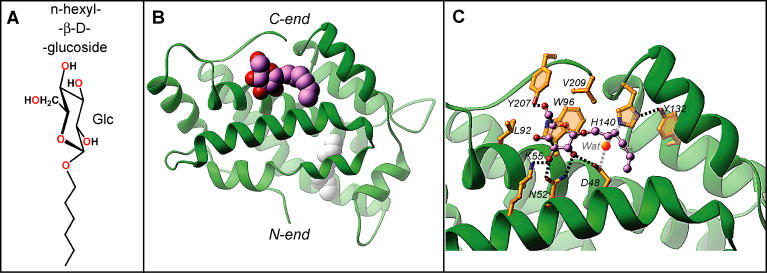
Structure of the *n*-hexyl-β-d-glucoside-GLTP Complex (A) Chemical formula of *n*-hexyl-β-d-glucoside. (B) Crystal structure of the *n*-hexyl-β-d-glucoside-GLTP complex, with the *n*-hexyl-β-d-glucoside molecule accommodated within the sugar recognition center on the GLTP surface. The GLTP is shown in a green ribbon representation, and the carbon atoms of the *n*-hexyl-β-d-glucoside are shown in a lavender space-filling representation. Extraneous hydrocarbon is shown in a white space-filling representation. (C) The *n*-hexyl-β-d-glucoside interactions with GLTP recognition center residues. Hydrogen bonds are shown by dashed lines. The bound ligand atoms are colored by lavender, red, and blue for carbon, oxygen, and nitrogen atoms, respectively. The water molecule bridging H140 with D48 is shown by bright red sphere.

To search for additional conformational states related to the GLTP working cycle, we cocrystallized GLTP with a model compound, *n*-hexyl-β-d-glucoside (Figure 5A), in which the sugar headgroup was β-linked to a single short (six-carbon) hydrocarbon chain, rather than a two-lipid chain ceramide moiety. GLTP binds the entire ligand at the protein surface (Figure 5B), with the hydrophobic tunnel remaining closed and containing extraneous hydrocarbon as reported previously for apo-GLTP [16]. The sugar headgroup of *n*-hexyl-β-d-glucoside binds the GLTP recognition center in a manner similar to all GSL-GLTP complexes reported previously [16] and in this paper. The same network of multiple specific interactions are observed between the *n*-hexyl-β-d-glucoside headgroup and the GLTP recognition center (Figure 5C), with the only differences being the following: (1) the absence of hydrogen bonding between K55 and the glucosyl OH4 group because of its equatorial positioning and (2) the loss of hydrogen bonds involving ligands carrying the ceramide amide group and residues H140 and D48, which are now water bridged to each other (Figure 5C). Similar water-bridged hydrogen bonds between residues H140 and D48 have also been found previously in apo-GLTP [16]. Further, attempts to cocrystallize GLTP with simple sugar compounds (galactose and maltose) have been unsuccessful to date.

The superimposed structures of apo-GLTP (colored red), the hexyl-β-d-glucoside-GLTP complex (colored gold), and the 24:1 GalCer-GLTP complex (colored green) are compared in stereo in Figure 4A.

### Sedimentation Studies

**Figure 6:**
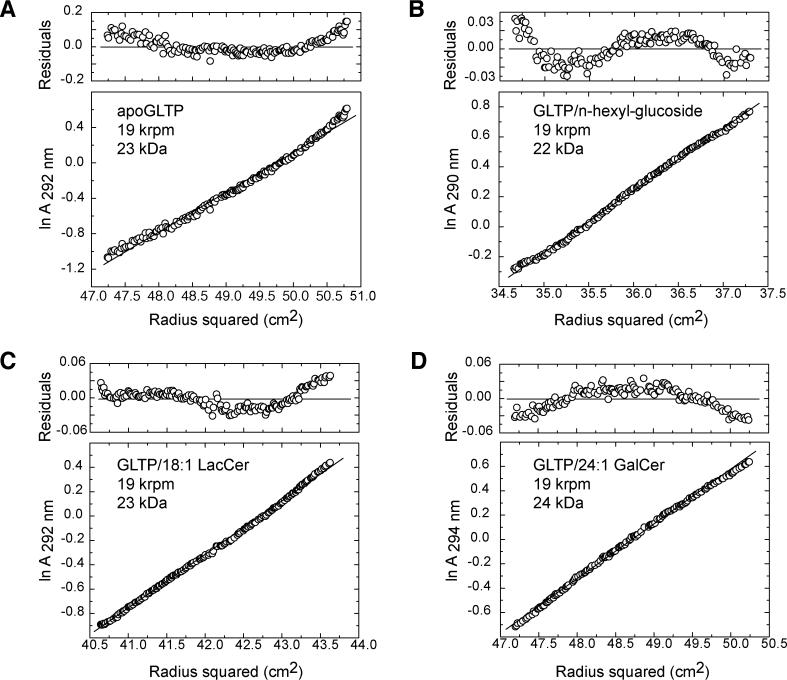
Analytical Ultracentrifugation Data on Apo-GLTP and its GSL Complexes. Analytical ultracentrifugation data of apo-GLTP (A) and GLTP complexes with *n*-hexyl-β-d-glucoside (B), 18:1 LacCer (C), and 24:1 GalCer (D). The 30 μM protein samples were centrifuged at 20 °C and 19,000 rpm in 20 mM Tris-HCl (pH 8.0) and 150 mM NaCl. Note that nonrandom residuals were observed in all cases.

Analytical ultracentrifugation measurements were used to determine the oligomerization state of GLTP in the absence and presence of GSL ligands. The apparent molecular masses of GLTP (Figure 6A) and its complexes with *n*-hexyl-β-d-glucoside (Figure 6B), 18:1 LacCer (Figure 6C), and 24:1 GalCer (Figure 6D) are in the 22–24-kDa range, consistent with apo-GLTP and its GSL complexes existing as monomers in solution. Analysis of residual differences from the monomeric model in each case reveals a systematic error, indicating that apo-GLTP and its GSL complexes are prone to aggregate in solution. Similar indications of monomeric solution behavior for GLTP, when liganded with 24:1 GalCer or in a GSL-free state, were obtained by size-exclusion chromatography and by affinity-tag immunoadsorption experiments (unpublished data).

### Crystal-Related Dimerization Interface in GSL-GLTP Complexes

**Figure 7:**
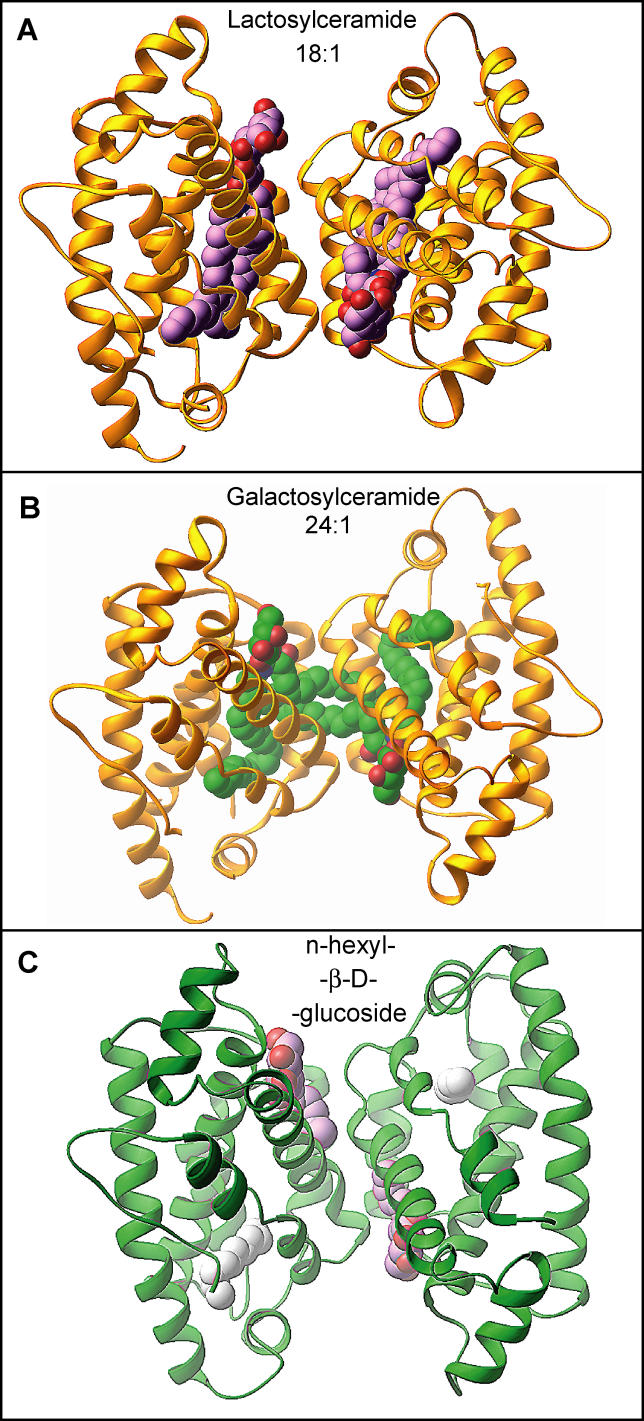
Crystal-Related Dimerization of GLTP Complexed with Different GSL Ligands (A) Crystal-related dimer in the structure of the 18:1 LacCer-GLTP [16]. The GLTPs are shown in a gold ribbon representation, and the carbon atoms of the LacCers are shown in a lavender space-filling representation. (B) Crystal-related cross dimer in the structure of the 24:1 GalCer-GLTP complex. The GLTPs are shown in a gold ribbon representation, and the carbon atoms of the GalCers are shown in a green space-filling representation. (C) Crystal-related dimer in the structure of the *n*-hexyl-β-d-glucoside-GLTP complex. The GLTPs are shown in a green ribbon representation, while the carbon atoms of the *n*-hexyl-β-d-glucoside are shown in a lavender space-filling representation. Extraneous hydrocarbon is shown in a white space-filling representation.

We have previously shown that apo-GLTP and its D48 mutant crystallizes in the *P2*_1_ space group (*a* = 55.4 Å, *b* = 35.3 Å, *c* = 57.5 Å, β = 115.8), whereas the LacCer-GLTP complex crystallizes in the *C2* space group (*a* = 75.6 Å, *b* = 49.1 Å, *c* = 68.5 Å, β = 122.5) [16]. All GSL-GLTP complexes reported in the present study crystallize in the *C2* space group, and exhibit unit cell parameters similar to those observed in the LacCer-GLTP complex [16], whereas the complex with *n*-hexyl-β-d-glucoside crystallizes nonisomorphously, with GSL-GLTP complexes and exhibits a doubling of the asymmetric unit volume (Table 1). Apo-GLTP and its D48V mutant are monomeric in the crystalline state [16]. By contrast, all the GSL-GLTP complexes, including the complex with *n*-hexyl-β-d-glucoside, exhibit crystal-related dimerization (Figure 7). The isomorphous nature of the crystals formed by the 18:1 LacCer-GLTP and 24:1 GalCer-GLTP complexes is noteworthy because their crystal-related dimerization interfaces display identical protein-protein arrangements, even though their sphingosine conformations differ substantially (Figure 7A and 7B). The dimerization interface generated upon crystallization enables clear and unequivocal resolution of the entire sphingosine chain from uninterrupted electron density maps (Figure 1D), even when the sphingosine is not fully encapsulated in the hydrophobic tunnel; i.e., 24:1 GalCer-GLTP.

**Figure 8:**
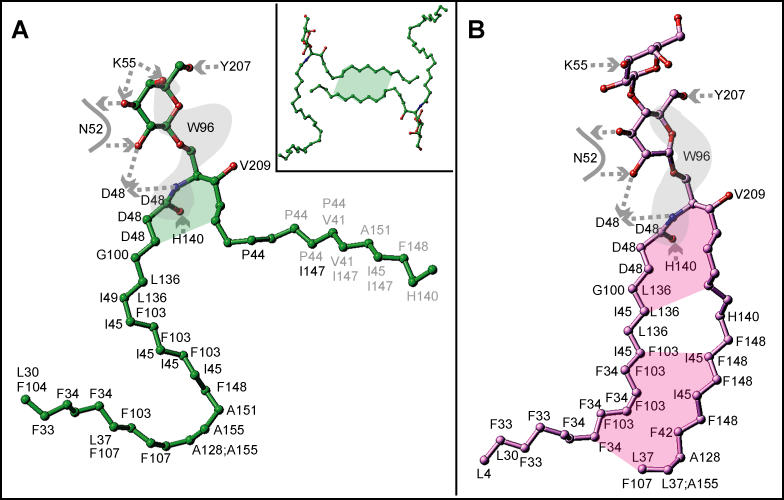
Comparison of the GLTP-Bound GSL Structures (A) Schematic of GSL interactions involving 24:1 GalCer in the sphingosine-out binding mode. Lettering indicates interacting GLTP amino acids, dashed arrows show hydrogen bonds oriented from donor to acceptor, the gray surface covers lipid atoms interacting with W96 indole group, the colored planes cover lipid regions participating in interchain interaction, gray lettering corresponds to interactions with a neighbor GLTP in the packing-related dimer in the crystal. The insert shows a schematic of sphingosine-sphingosine interaction of 24:1 GalCer in the crystal-related cross dimer. (B) Schematic of GSL interactions involving 18:1 LacCer in the sphingosine-in binding mode. Lettering and colored planes are defined as in (A).

The GLTP amino acid residues making the closest van der Waals contacts with the GSL chains for sphingosine-out and sphingosine-in alignments are outlined in Figure 8A and 8B, respectively. The sphingosine-in mode is additionally stabilized by acyl-sphingosine interchain interactions (Figure 8B), whereas in the sphingosine-out mode, sphingosine-sphingosine interactions between GSLs from partner complexes associated with crystal-related cross dimerization contribute to stabilization (Figure 8A, inset). The terminal methyl groups of each of the two sphingosine chains enter into the hydrophobic tunnels of their partner GLTP molecules (Figure 7B), filling unoccupied tunnel space in the cross dimers observed for crystals of the complex.

### GSL-Induced Variations of GLTP Surface Interaction Propensities

To independently evaluate the interaction propensity of the surface regions of GLTP, in both its apo- and GSL-complexed forms, we applied the optimal docking area (ODA) algorithm [29] to identify regions on the protein surface that display a favorable energy change upon desolvation. The calculated energy changes reflect the effect of replacing the water environment for a lower dielectric (but still polar) medium, such as the surface of another protein or a membrane.

**Figure 9:**
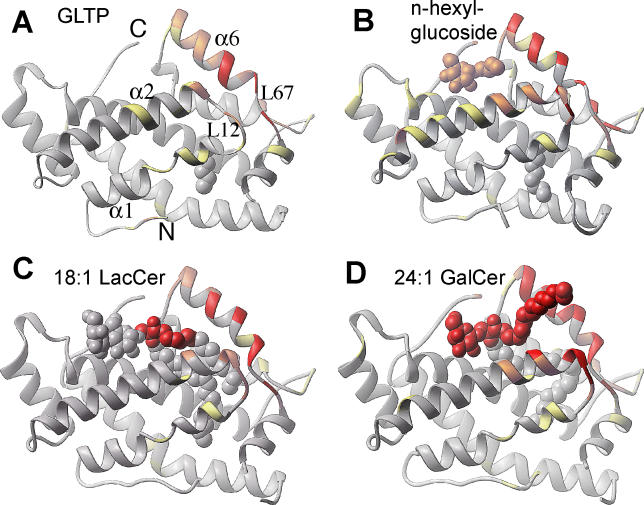
Distribution of the Protein-Protein and Protein-Membrane Interaction Propensities over the GLTP Surface Calculated by the ODA Approach for Four Different GLTP Conformational States (A) apo-GLTP with an extraneous hydrocarbon. (B) GLTP complex with *n*-hexyl-β-d-glucoside. (C) Sphingosine-in GLTP complex with 18:1 LacCer. (D) Sphingosine-out GLTP complex with 24:1 GalCer. Structures are colored by the absolute magnitude of the ODA signal from the strongest in red, through medium in orange and weak in yellow, to the weakest in gray.

The ODA-based calculated distribution of protein-protein (protein-membrane) interaction propensities over the GLTP surface for apo-GLTP is shown in Figure 9A, and those for GLTP complexed with *n*-hexyl-β-d-glucoside, 18:1 LacCer (sphingosine-in alignment), and 24:1 GalCer (sphingosine-out alignment) are shown in Figure 9B, 9C, and 9D, respectively (corresponding crystal-related dimerization interfaces are shown for the complexes in Figure S3). Structures are colored by the absolute magnitude of the ODA signal (red, strongest; orange, medium; yellow, weak; gray, weakest). The first observation is that the area corresponding to maximum surface interaction propensity—the spanning α helix 6, the loop L12, the N terminus of α-helix 2, and the loop L67—represents the only common ODA patch spanning all four structures (Figure 9A–9D). This common ODA patch coincides with structural elements that were previously proposed to constitute the cleft-like GLTP gate governing GSL binding/release [16], as well as with the crystal-related dimerization interfaces (Figure S3A–S3D). This finding suggests that the ODA patch has an enhanced propensity for interaction with proteins (associated with dimerization interface in the crystal) and/or membranes (associated with GSL binding/release). Another observation is that the intensity of the ODA signal differs between structures in Figure 9A–9D, ranging from a weak signal for the apo-form (Figure 9A) to a very strong signal for the sphingosine-out complex (Figure 9D).

To evaluate the potential of the intermolecular interaction in more quantitative terms, we computed the change in accessible surface area that results when the contact interface of the crystal-related dimer is created from the two monomers, thereby yielding computed values of the dimerization free energy for the four classes of structures outlined in Figure 9A–9D. These values, together with the ODA propensity, are shown in Table 2 and highlight GSL-induced variations of GLTP surface interaction propensities.

**Table 2:**
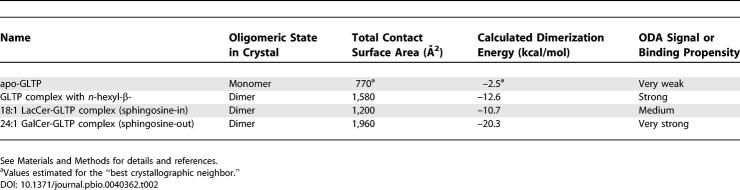
Calculated Parameters Characterizing the Surface Interaction Propensities of Different GLTP States

### Structure of the GSL-GLTP Complex Containing Short Acyl Chain GSLs

**Figure 10:**
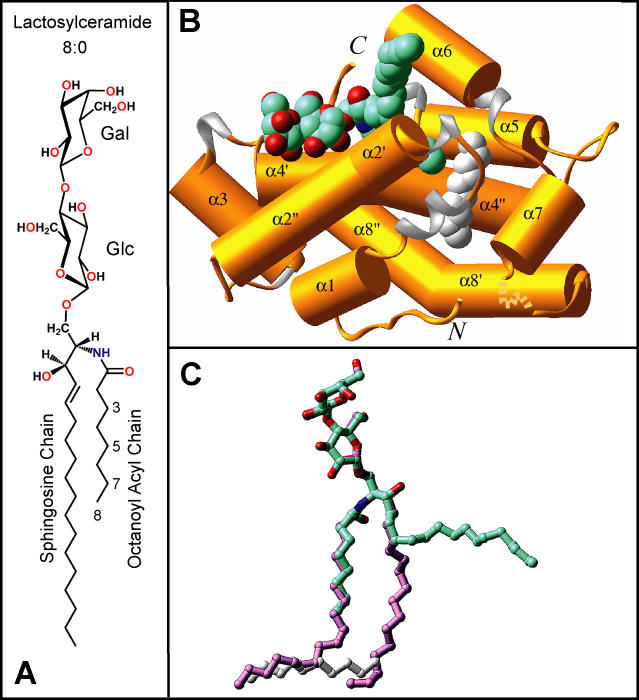
Structure of the 8:0 LacCer-GLTP Complex (A) Chemical formula of 8:0 lactosylceramide. (B) Crystal structure of the 8:0 LacCer-GLTP complex. The GLTP is shown in a gold ribbon representation, and the carbon atoms of the LacCer are shown in a cyan space-filling representation. (C) Superposition of stick representations of the 8:0 LacCer (carbon atoms colored in cyan) and 18:1 LacCer (carbon atoms colored in lavender) derived from their sphingosine-out and sphingosine-in GLTP complexes, respectively.

We have proposed above that the sphingosine-out binding mode of 24:1 GalCer in its GLTP complex can be attributed to obstruction of the sphingosine chain from assuming a chain-parallel orientation within the channel by the serpentine trajectory of the 24-carbon nervonoyl acyl chain. Assuming that this proposition is correct, we hypothesized that GSLs with short acyl chains should occupy the channel in a similar fashion as 18:1 LacCer. To test this hypothesis, we cocrystallized GLTP with LacCers containing 8:0 octanoyl (Figure 10A) and 12:0 dodecanoyl acyl chains. The use of these two LacCer species, which have shorter acyl chains than LacCers in mammalian cells, provided an investigative tool to gain insights into how GLTP accommodates GSLs with different acyl structures.

Surprisingly, the binding mode of 8:0 LacCer to GLTP (GSL colored cyan in Figure 10B) resembles that of the 24:1 GalCer-GLTP complex (GSL colored green in Figure 1B), rather than 18:1 LacCer-GLTP complex (GSL colored lavender in Figure S2B). This outcome occurred despite the octanoyl acyl chain being short enough so as not to hinder the sphingosine chain from occupying the hydrophobic tunnel. The sphingosine-out alignment results as a consequence of the narrow bottom of the hydrophobic channel being occupied by a short extraneous hydrocarbon chain (colored silver in Figure 10B and 10C). In the 8:0 LacCer-GLTP complex, the extraneous hydrocarbon chain consists of ten carbons (Figure 10B and 10C), so it would compete for the tunnel region with the sphingosine chain if the latter took the “in” position. Therefore, the sphingosine chain bends, and stays outside (cyan colored lipid chain in Figure 10C), and can be observed bridging to the tunnel of the neighboring GLTP in the crystal-related cross dimer (Figure S4A).

Similar behavior was observed in the 12:0 LacCer-GLTP complex, with an extraneous hydrocarbon being found in the same location as in the 8:0 LacCer-GLTP complex. In the case of 12:0 LacCer, the five terminal carbons of the dodecanoyl acyl chain are disordered in the crystal, suggesting insufficient hydrocarbon length to displace the extraneous hydrocarbon, which blocks the sphingosine chain from entering the tunnel. The net result is outside positioning of more than half of the sphingosine chain and bridging to the tunnel of the partner GLTP in the crystal-related cross dimer.

### Influence of *cis* Double Bonds on Acyl Chain Conformation within the GLTP

We previously reported on the structure of 18:1 LacCer-GLTP complex, where the 18-carbon oleoyl acyl chain contained a single *cis*-9,10 double bond (Figure S2A) [16]. Because the number and positioning of double bonds in the acyl lipid chain could influence its conformational flexibility, GLTP was also cocrystallized with 18:2 GalCer containing a linoleoyl acyl chain with *cis*-9,10 and *cis*-12,13 double bonds (18:2^Δ9,12cis^) (Figure S5A).

The structure of the 18:2 GalCer-GLTP complex is shown in Figure S5B. The 18-carbon linoleoyl acyl chain adopts a serpentine trajectory due to the restricted rotation about the *cis*-9,10 and *cis*-12,13 double bonds (Figure S5A), thereby preventing the chain from entering the narrow bottom segment of the hydrophobic channel, which in any case is occupied by an extraneous five-carbon hydrocarbon chain (colored silver in Figure S5B and S5C). As a result, the terminal part of the linoleoyl acyl chain (longer lemon colored lipid chain in Figure S5C) occupies the tunnel region that would have been occupied by the sphingosine chain. The sphingosine chain (shorter lemon colored lipid chain in Figure S5C) bends and projects outwardly, enabling insertion into the neighboring GLTP in the crystal-related cross dimer, and promoting the same structural arrangement of the 18:2 GalCer-GLTP complex (Figure S5B), as has been observed in the 24:1 GalCer-GLTP complex (Figure 1B) and the 8:0 LacCer-GLTP complex containing extraneous hydrocarbon (Figure 10B).

Although the acyl lipid chain length is the same in 18:1 LacCer (Figure S2A) and 18:2 GalCer (Figure S5A), their ceramide lipid chains adopt different conformations inside the GLTP tunnel, with a sphingosine-in conformation in the former complex (lavender colored shorter lipid chain in Figure S5C) and a sphingosine-out conformation in the latter complex (lemon colored shorter lipid chain in Figure S5C).

### Comparison of GSL Positioning within the GLTP

**Figure 11:**
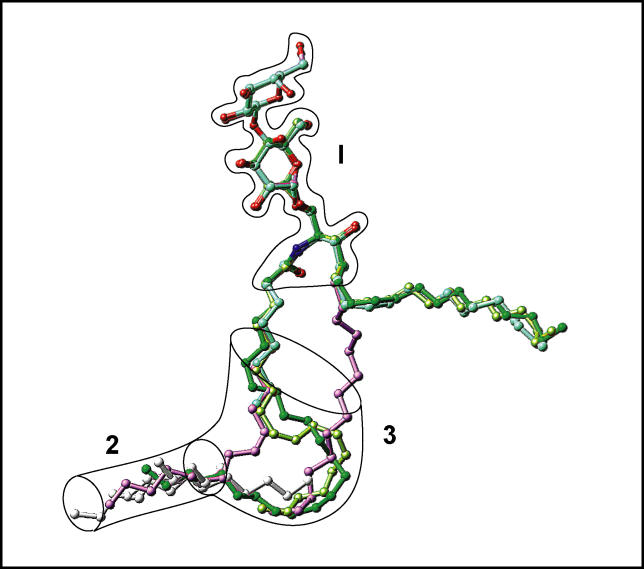
Schematic Highlighting Positions of Lipid Chains and Extraneous Hydrocarbons in GSL-GLTP Complexes The assembly of bound glycolipids and extraneous hydrocarbons, if present in the GLTP tunnel, are shown. The bordered segment labeled 1 encompasses the sugar- and amide-binding site on the GLTP, whereas bordered segments 2 and 3 span lipid-binding sites within the hydrophobic GLTP tunnel. The narrow bottom of the GLTP tunnel is schematically represented by a transparent cylinder, labeled 2. The segment labeled 3 is collapsed in apo-GLTP and its complex with *n*-hexyl-β-d-glucoside. The glycolipid atoms are colored red and blue for oxygen and nitrogen atoms, respectively, and by specific colors for carbon and extraneous hydrocarbons. Specific colors are green for 24:1 GalCer, cyan for 8:0 LacCer, lavender for 18:1 LacCer, lemon for 18:2 GalCer, and silver for extraneous hydrocarbons accompanying 8:0 LacCer, 18:2 LacCer, and apo-GLTP. The longest extraneous hydrocarbon, which is the only one entering the region labeled 3, accompanies 8:0 LacCer.

All GLTP-bound GSLs examined to date are displayed together in a single composite overlay in Figure 11, along with the position of the relevant extraneous hydrocarbons. The sphingosine-in binding mode is characteristic of the 18:1 LacCer complex, whereas the remaining GSLs adopt the sphingosine-out binding mode, in several cases facilitated by extraneous hydrocarbon (Figure 11). The resulting overview of superimposed GSL structures shows which regions of the GLTP liganding site can adapt to the structural variation of the ligand and which parts of the GLTP liganding site are fixed in an invariant way when interacting with glycolipid ligand. There are two invariant regions, designated 1 and 2, as shown in Figure 11. One of these, region 2, is indicated by a cylinder, delineating the narrow bottom compartment of the hydrophobic tunnel; the other, region 1, is represented by the outlined glycolipid sugar-amide segment. Region 3, which is a continuation of invariant region 2, is collapsed in apo-GLTP and its complex with *n*-hexyl-β-d-glucoside, but it forms, together with region 2, the GLTP hydrophobic tunnel, which encapsulates GSL lipid chains at the complex level.

Comparison of GLTP liganding affinity for the different GSLs revealed the following similar dissociation constant (*K*_d_) values: 18:1 LacCer (0.27 μM); 24:1 GalCer (0.25 μM); 8:0 LacCer (0.2 μM), and 18:2 GalCer (0.2 μM). These values were estimated from changes in the tryptophan fluorescence; i.e., decrease in emission intensity (≈40%) and strong blue shift of the emission wavelength maximum (≈14 nm) that are known to occur on interaction with porcine brain GalCer, 18:1 GalCer [30], and palmitoyl GalCer [31].

## Discussion

### Overview

Our previous research on the structures of apo-GLTP and its complex with LacCer highlighted the conformational transition generating a hydrophobic channel on GSL complex formation [16]. Both the acyl and sphingosine lipid chains were inserted into a hydrophobic channel in the LacCer-GLTP complex in a parallel-like alignment (Figure S2B and S2C). It was proposed that GSL incorporation/release was most likely mediated by a gate-like mechanism, thereby providing access to the hydrophobic tunnel. Here we expand on the previous observation of a sphingosine-in structure of the 18:1 LacCer-GLTP complex to reveal sphingosine-out structures with GSLs of varying acyl chain length and double-bond character. Because the sphingosine-out conformation can be directly observed in four out of five of our GLTP-GSL complexes and is implicated in a recently reported GM3-His_6_–tagged bovine GLTP complex, in which the last ten of 18 carbons of the GM3 sphingosine chain are unobservable because of disorder [28], the sphingosine-out conformation appears to be preferred by GLTP. Our results establish a structural basis for the accommodation of various GSL species with different acyl chemical structures by GLTP and provide support for a concerted sequence of events during GSL acquisition and/or release.

### GSLs in GLTP Recognition Center

The invariant nature of the sugar-amide recognition center of GLTP (outlined region 1 in Figure 11) results from the highly conserved noncovalent interactions that anchor the headgroups of different glycolipids to the protein surface. The galactose group of bound GalCers forms all hydrogen bonds and displays all van der Waals contacts (Figure 2A) that were previously observed for the sugar residue adjacent to ceramide in bound LacCers [16]. The ceramide amide moiety is also similarly positioned in all complexes (Figure 11) through a pair of hydrogen-bonding interactions with D48 and H140, with their alignment facilitated by hydrophobic contacts with V209, as noted for the 24:1 GalCer-GLTP complex (Figure 2A). Thus, all hydrogen bonds and van der Waals interactions found between the GLTP recognition center and the sugar-amide headgroup in GSL are conserved, except for hydrogen-bond interactions involving the K55 ɛ-amino group (Figure 2A) [16]. Point mutations support the key role of W96, H140, D48, and N52 in the recognition and anchoring of the sugar-amide moieties by GLTP [16,26], because mutations W96A and H140L result in almost complete inactivation, and mutations D48V and N52I result in significant inactivation. On the other hand, despite the hydrogen bonding by residues K55 and Y207 with the glycolipid headgroups in all complexes, mutations K55I and Y207L retain almost full transfer activity, as measured by both galactosyl- and lactosylceramide transfer-binding assays [16,32]. These findings lead us to propose two categories of interactions: (1) primary, which are invariant and involve the recognition and anchoring of the first sugar and amide group; and (2) secondary, which serve a less essential supporting role and provide flexibility for binding of more complex sugar headgroups. Examples of the latter are K55 and Y207, which appear likely to play roles in the liganding of other GSLs known to be transferred by GLTP [12,32,33]. Lysine K55 appears to be a good candidate to interact specifically with negatively charged glycolipid head groups, such as sulfatide or gangliosides; the phenyl ring of Y207 appears to be a good candidate for undergoing a stacking interaction with extended and/or branched sugars of complex GSLs. This stacking interaction would be analogous to that of W96 with the initial sugar of GalCer (Figure 2A) and LacCer [16], observed in our GSL-GLTP complexes.

The all–α-helical conformation of GLTP does not prevent its sugar-amide recognition center from sharing two fundamental features of the liganding centers of many other carbohydrate binding proteins including all known galactose-specific binding proteins, which display α + β or all-β conformations. The two fundamental features are: (1) hydrogen bond donor/acceptor residues arranged so as to facilitate interactions with hydroxyl groups along the perimeter of the pyranose ring and (2) the presence of an aromatic residue, i.e. tryptophan, against which the sugar stacks [34,35]. Our observation of decreased glycolipid transfer activity in the W96F mutant of GLTP [16] is consistent with the idea that the larger stacking platform provided by tryptophan (compared to phenylalanine or tyrosine) facilitates positional orientation of the sugar for optimal hydrogen bonding interactions with the perimeter residues [36]. It is also noteworthy that all hydrogen bond classes observed in previous protein-carbohydrate interactions [34] are evident when GLTP interacts with the GSL sugar-amide. Among the classes are bidendate hydrogen bonding involving N52, bifurcated hydrogen bonds involving D48 (and K55 in case of galactose), as well as the propensity of the OH2 and OH3 sugar hydroxyl groups and amino acid side chains (e.g., Y207) to form cooperative hydrogen bonds.

### Two Compartments within the GLTP Hydrophobic Tunnel

The upper part of the hydrophobic tunnel (region 3 in Figure 11) is collapsed in apo-GLTP (Figure 3A) and when GLTP is complexed with *n*-hexyl-β-d-glucoside. The solvent-accessible volume (evaluated by the program Computed Atlas of Surface Topography of proteins [CASTp]: http://cast.engr.uic.edu) of the hydrophobic tunnel which is ~320 Å^3^ in the sphingosine-in complex and ~230–270 Å^3^ in the sphingosine-out complexes, drops to 100–170 Å^3^ in apo-forms (native and D48V mutant) and 140 Å^3^ in complex with *n*-hexyl-β-d-glucoside. In apo-GLTP and the *n*-hexyl-β-d-glucoside–GLTP complex, the tunnel has collapsed, although the bottom (represented by cylinder 2, Figure 11) remains occupied by an extraneous hydrocarbon (Figures 3A, 5B, and S1A).

The upper compartment of the hydrophobic tunnel, when open via its cleft-like gate, can accommodate the two hydrocarbon chains of ceramide in a side-by-side alignment, under the strict condition of a very tight fit with the walls of the tunnel (Figure 3B). Such a tight fit has been observed in the 18:1 LacCer-GLTP complex [16] and is characteristic of the sphingosine-in binding mode, which represents the accommodation limit for ceramide encapsulation.

During the process of glycolipid acquisition by GLTP, the glycolipid acyl chain must be sufficiently long and flexible so as to occupy the upper compartment (Figure 11, region 3) and enter the narrow bottom compartment (Figure 11, region 2) of the tunnel to displace the extraneous hydrocarbon chain. If sphingosine entry into the upper tunnel compartment is obstructed by either the presence of extraneous hydrocarbon or by steric hindrance created by the acyl chain itself, then the sphingosine chain stays out. Thus, both compartments of the hydrophobic tunnel play critical and essential roles in accommodating the acyl and sphingosine chains of ceramide and determining the final equilibrium localization of the sphingosine chain.

Disordering of the GLTP N terminus provides a potential mechanism for controlling access to the hydrophobic tunnel via a portal located in the narrow bottom compartment (Figure S1B). N-terminal disorder, observed in all our structures, decreases considerably when GLTP complexes GSL. The net result is that leucine L4 is positioned to act like a cork to plug the entrance to the portal. It is noteworthy that stacking (3.4 Å separation) between histidine H7 of the N terminus and H29 at the beginning of the 1–2 loop (between helices 1 and 2) is observed in all our GSL-GLTP complexes. The ordering effect of this stacking interaction on the N terminus of GLTP appears to enable the corking action of L4, which is further stabilized by van der Waals interactions with the terminal methyl of the GSL acyl chain near the channel bottom. A disruption of the H7/H29 stacking is observed in all apo-GLTP structures in which the H7 residue is resolved, including in our D48V apo-GLTP, as well as in our *n*-hexosyl glucoside–GLTP complex. We suggest that the presence of a His_6_ fusion tag—i.e., RGSHHHHHHGS— such as that used in recent studies of a GM3-His_6_-tagged bovine GLTP complex, could enhance disorder in the N terminus and prevent L4 from corking the open portal, thereby facilitating unobstructed passage of long GSL acyl chains completely through the hydrophobic channel [28].

Other differences between the structures of all of our GSL-GLTP complexes and the GM3-His_6_-GLTP complex [28] include the lack of outward movement of helix 6 and lack of swinging out of F148 at the hydrophobic tunnel entrance in the GM3-His_6_-GLTP (relative to apo-GLTP).

### Acyl Chain Structure Is a Key Determinant of Ceramide Conformation in GLTP

Of the two hydrocarbon chains of the GSL ceramide moiety, it is the structure and conformational flexibility of the acyl chain, rather than the sphingosine chain, that plays the dominant role in determining how the GSL is accommodated by the GLTP. Contributing to this dominance by the acyl chain is the proposed oriented entry of the ceramide through the cleft-like gate, most likely necessitating that GLTP accommodate the acyl and sphingosine chains in sequential fashion. The sphingosine-in mode is favored when the acyl chain is just long and flexible enough to enter the narrow bottom (region 2) of the channel and fill it without creating steric hindrance for entry of the sphingoid chain. In other words, both chains fit optimally into the available channel space (Figure 3B).

The sphingosine-out mode is favored for GSL with long acyl chains (>19 carbons), because the tunnel length is limited and the acyl chain must bend in serpentine fashion to be accommodated within the channel (Figure 3C). In this case, sphingosine entry into the same channel is obstructed. Short acyl chains (<13 carbons) are not long enough to enter the narrow bottom compartment of the GLTP hydrophobic channel and displace embedded extraneous hydrocarbon chain. The result is steric hindrance to the entry of sphingosine into the channel, thereby favoring the sphingosine-out binding mode (Figure 10B and 10C). Thus, there is a limited range of chain lengths that enable a stable, tight fit of the acyl chain in the GLTP tunnel without interfering with the entry of the sphingosine chain, and ensuring a sphingosine-in binding mode.

In essence, the sphingosine-out conformer in GSL-GLTP complexes is analogous to monoacylation of a soluble protein, except that the exposed hydrocarbon segment of sphingosine (10–12 methylenes) is even shorter than protein acylation achieved by myristoylation (14:0) or by palmitoylation (16:0) events. It is well established that attachment of a single myristoyl group to a soluble protein only marginally affects its partitioning affinity for nonpolar (membrane) surfaces [37]. Generally, a second acylation event involving palmitate (16 carbons) is needed for soluble proteins to form strong, stable associations with membranes. Also, there are examples of proteins containing surface grooves that accommodate significant stretches of nonpolar hydrocarbons on their surfaces while remaining monomeric [38,39]. Thus, it is not surprising that GLTP and its GSL-bound complexes are monomeric in solution, as monitored by sedimentation analysis or size-exclusion chromatography, even when a substantial portion of the GSL sphingosine chain (10–12 methylenes) is outside the hydrophobic tunnel.

### Extraneous Hydrocarbons

It should be emphasized that in all our crystal structures, the narrow bottom of the GLTP hydrophobic tunnel (depicted by narrow cylinder, labeled 2 in Figure 11) is always occupied, either by the glycolipid acyl chain termini (complexes with 18:1 LacCer and 24:1 GalCer) or by extraneous hydrocarbons (complexes with 12:0 and 8:0 LacCers, 18:2 GalCer, *n*-hexyl-β-d-glucoside, and GSL-free GLTP). Even in apo-GLTP ([16], Figure S1A) and in the *n*-hexyl-β-d-glucoside-GLTP complex (Figure 5B), in which the upper part of the hydrophobic tunnel is collapsed, the narrow bottom is occupied by extraneous hydrocarbons. As pointed out previously, it is not clear if the extraneous hydrocarbon is acquired during heterologous expression in E. coli or subsequent crystallization*.* However, the situation is not surprising for GSL binding proteins [22] and has been observed with other lipid binding/transfer proteins [20,25]. Characterization in the published literature of these extraneous lipids has revealed a variety of lipid and hydrocarbon structures. In any case, our structures indicate that the bottom of the hydrophobic tunnel (region 2 in Figure 11) is an essential feature and therefore occupied in all available structures by a tightly-fitted nonpolar lipid or extraneous hydrocarbon chain.

To determine whether the extraneous lipid can be removed from the hydrophobic channel, apo-GLTP was incubated with β-methylcyclodextrin because of its established ability to bind a variety of nonpolar substances including different lipids. Structural characterization showed that the extraneous lipid still remained within the hydrophobic channel (unpublished data), consistent with a relatively high affinity for occupancy by at least one nonpolar chain. For the purposes of the present study, the strong affinity of the GLTP hydrophobic channel for occupancy by extraneous lipid has provided an investigative tool to evaluate the structural basis for the accommodation of various GSL species with different acyl chemical structures by GLTP and a tool to show that the acquisition and/or release of glycolipid proceeds in an adaptive manner via highly oriented, concerted, and sequential events.

### GLTP Conformational States along the GSL Acquisition Pathway

The conformation of the L12 loop, located between α helices 1 and 2 of GLTP, appears to be especially responsive to point mutations and ligand binding. Thus, the conformation of the L12 loop differs between apo-GLTP (colored red, Figure 4A) and the GLTP complex with *n*-hexyl-β-d-glucoside (colored gold, Figure 4A), even though the upper part of the hydrophobic tunnel (segment 3, Figure 11) is collapsed in both cases. Loop L12 in the D48V mutant of apo-GLTP [16], where D48 (located on α helix 2) of the recognition center is replaced by hydrophobic valine, adopts yet another conformation (unpublished data), which is intermediate between the native apo-form and the complex with *n*-hexyl-β-d-glucoside. Taken together, these data indicate that events in the GLTP headgroup recognition center contribute to the conformation of the L12 loop.

A comparison of the conformation of the L12 loop in GLTP complexes with *n*-hexyl-β-d-glucoside (colored gold, Figure 4A) and 24:1 GalCer (colored green, Figure 4A), indicates that ligand-sugar attachment facilitates adoption by this loop of the conformation approaching that observed in GSL-GLTP complexes. In addition, on GSL acquisition, α helix 2 moves along its axis, with an outwards movement of both its N-terminal segment and adjacent loop L12, while α helix 6 moves outwards by ≈2.7 Å (Figure 4A, transition from gold to the green-colored conformations). The latter movements accompany the opening of the hydrophobic tunnel associated with acquisition and accommodation of the acyl chain. The subsequent acquisition of the sphingosine chain associated with the sphingosine-in complex, should it occur, results in an expansion of the existing hydrophobic tunnel from the volume of 230–270 Å^3^ to 320 Å^3^. In contrast, on *n*-hexyl-β-d-glucoside acquisition, there is only limited outwards movement of the α helix 2 N-terminal segment and adjacent loop L12, but no movement of the α helix 2 along its axis nor outward movement of α helix 6, keeping phenylalanine F148 in a swung-in position and allowing for only partial opening of the hydrophobic channel, in a fashion similar to that observed recently in GM3-His_6_-tagged bovine GLTP complex [28].

### GSL-Based Distribution of GLTP Surface Interaction Propensities

Monomeric apo-GLTP, which has the lowest total contact surface area of 770 Å^2^, exhibits the smallest computed dimerization energy of −2.5 kcal/mol, whereas the 24:1 GalCer-GLTP complex, which has the highest total contact surface area of 1,960 Å^2^, exhibits the largest computed dimerization energy of −20.3 kcal/mol (Table 2). The *n*-hexyl-β-d-glucoside-GLTP complex exhibits somewhat larger values of total contact surface area and computed dimerization energy than the 18:1 LacCer-GLTP complex. The different oligomeric states of a ligand-free GLTP (monomer in solution and crystal) and sphingosine-in and sphingosine-out GLTP complexes (monomer in solution; packing-related dimerization in crystal) might reflect a gain of protein-protein or protein-membrane interaction propensities by the protein surface upon ligand binding (Table 2).

Only monomeric GSL-GLTP complexes could be detected in solution despite repeated attempts using different methodologies (e.g., analytical ultracentrifugation, size-exclusion liquid chromatography, affinity pull-down assays involving GLTPs with different N-terminal fusion tags). This finding, when viewed within the context of the ODA energy contact calculations, implies that the GLTP face in contact with its partner protein in the crystal-related dimer identifies this dimerization interface as the likely membrane interaction site of GLTP, because it is rather hydrophobic, is ringed by four lysines, and contains several tyrosines and two tryptophan residues, which are all known to provide a favorable interaction site with membranes [31,40–42].

### Model of GSL Transfer/Presentation by GLTP

Our findings of a new sphingosine-out ligand binding mode in the crystal structures of GSL-GLTP complexes, the different conformational changes induced in the gate-region L12 loop upon liganding of different glycolipids, and the respective computed values characterizing the interaction propensities are collectively supportive of an important property of GLTP: namely, a highly conserved, adaptive, and concerted sequence of events involving GLTP-mediated acquisition/release of GSL. Because apo-GLTP displays a relatively weak protein/membrane-binding propensity (Table 2) [42], it can be expected to readily partition on and off the membrane surface. Nonetheless, the rapid lateral diffusion rates of lipids in fluid-phase membranes and the confinement of the glycolipid ligand to a membrane surface would likely enhance the capacity of GLTP to associate with a glycolipid molecule among other membrane lipids. The sugar moiety of the glycolipid clearly acts as a primary specificity determinant, whereas the ceramide amide functional group orients the entry of the hydrocarbon chain(s) through the cleft-like gate. Comparison of the apo-GLTP and the *n*-hexyl-β-d-glucoside–GLTP complex provides clear evidence that the recognition and anchoring of the sugar head triggers conformational rearrangements of the gate region, L12 loop. At this stage, there is an increased interaction propensity centered about the cleft-like GLTP gate that is surrounded by aromatic surface residues (e.g., W142, W96, Y153, Y157, Y207, Y81) and a half dozen lysine residues, which are known to interact favorably with membrane interfaces [40,41], thereby potentially facilitating gate-membrane interactions, including the outward movement of the α helix 6. We anticipate that the opening of the gate in the membrane-associated state facilitates entry of the acyl chain into the hydrophobic channel of the GLTP. The accommodation limits of the hydrophobic tunnel, illustrated by the crystal structures of the GSL-GLTP complexes, strongly suggest that sphingosine is the last lipid part to enter GLTP and, most likely, the first to depart GLTP upon interaction with a membrane.

### Functional Implications

Among known lipid binding/transfer proteins, the GLTP topology is unique. The GLTP fold also defines a novel membrane targeting and/or interaction domain among known peripheral proteins [27]. The formation of a lipid-liganding site by α-helical layering, without intramolecular disulfide bonds, contrasts with the situation in other lipid binding and transfer proteins which use either disulfide-bridge–stabilized, helical bundles (i.e., saposin folds), or motifs dominated by β sheets (i.e., β grooves or concave cups) and β barrels. Although the phosphatidylcholine and phosphatidylinositol transfer proteins appear to show a preference for liganding certain phosphoglyceride species during intermembrane transfer [43,44], our findings with GLTP establish a structural basis for the accommodation of various GSL species with different acyl chemical structures by the liganding channel of a lipid transfer/binding protein. The present structural analyses have provided insights into the specificity of glycolipid targeting by GLTP and also demonstrated how the accommodation limits of the GLTP liganding site are controlled by the conformational features of both the hydrophobic channel and the GSL ceramide lipid chains. Quite remarkably, in addition to the expected essential role of the sugar-amide headgroup recognition center in controlling the selectivity for glycolipids, we have found that the length and conformational flexibility of the glycolipid acyl chain constitute critical features modulating the accommodation of sphingosine chain. Finally, our observation of crystal-related cross dimerization in the crystal structures of several sphingosine-out GSL-GLTP complexes suggests that the ceramide lipid chains of GSLs most likely enter into and egress from GLTP in a defined stepwise manner.

## Materials and Methods

### Protein expression and purification.

The open reading frame (ORF) encoding human GLTP was subcloned into the pET-30 Xa/LIC expression vector (Novagen, Madison, Wisconsin, United States), enabling cleavage of the amino-terminal His_6_ and S-tags, as previously described [16]. Transformed BL21 (DE3) E. coli cells (Novagen) were grown in Luria-Bertani (LB) medium at 37 °C, induced with 0.1 mM IPTG, and grown an additional 20 h at 15 °C. Purification of recombinant GLTP (rGLTP) from soluble lysate protein was accomplished by Ni-affinity chromatography [32]. The His_6_-tag and S-tag sequences were removed from rGLTP by incubation with Factor-Xa at room temperature for 16 h. rGLTP was then purified by fast protein liquid chromatography size-exclusion chromatography and concentrated to 10–15 mg/ml in 150 mM NaCl, 20 mM Tris-HCl, pH 8.0.

### Glycolipid synthesis and purification.

LacCer and GalCer with homogeneous acyl chains were produced by reacylating d-lactosyl-β1–1′-d-*erythro*Sphingosine (lyso LacCer) and d-galactosyl-β1–1′-d-*erythro*Sphingosine (lyso GalCer or psychosine) (Avanti Polar Lipids, Alabaster, Alaska, United States) with the desired fatty acyl residue as described previously [45,46]. Briefly, the *N-*hydroxy succinimide ester of the desired fatty acid was prepared, recrystallized, and reacted with lyso-glycolipid. Reacylation was performed at 60 °C under nitrogen for 6–8 h in the presence of the catalyst, *N*-ethyldiisopropylamine. Following reacylation, the glycolipid was purified by flash column chromatography and crystallized from CHCl_3_/CH_3_OH using −20 °C acetone. Using the preceding approach, *N*-dodecanoyl lactosylsphingosine (12:0 LacCer), *N-*cis-9-octadecenoyl lactosylsphingosine (18:1^Δ9cis^ LacCer or *N*-oleoyl LacCer), *N-*cis-15-tetracosenoyl galactosyl-sphingosine (24:1^Δ15cis^ GalCer or *N*-nervonoyl GalCer), and *N-*cis-*N-*cis-9,12-octadecenoyl galactosyl-sphingosine (18:2^Δ9,12cis^ GalCer or *N*-linoleoyl GalCer) were prepared. *N*-octanoyl lactosylsphingosine (8:0 LacCer) was obtained from Avanti Polar Lipids. Acyl homogeneities of the glycolipid derivatives were confirmed by quantitative release, methylation, and analysis of the fatty acyl residues via capillary gas chromatography. Glycolipid purity was confirmed by thin layer chromatography. *n*-hexyl-β-d-glucoside was obtained from Anatrace (Maumee, Ohio, United States).

### Sedimentation equilibrium analysis.

Analytical ultracentrifugation measurements were carried out on a Beckman XL-A (Beckman Coulter, Fullerton, California, United States) analytical ultracentrifuge equipped with an An-60 Ti rotor (Beckman Coulter) at 20 °C. His_6_-tagged GLTP and His_6_-tagged GLTP complexes were loaded at initial concentration of 7, 15, and 30 μM and analyzed at rotor speed of 19,000 and 22,000 rpm. Data were acquired at wavelengths 290–294 nm and processed simultaneously with a nonlinear least-squares fitting routine [47]. Solvent density and protein partial specific volume were calculated according to solvent and protein composition, respectively [48]. We estimated the partial specific volume of GSLs as 0.98 mL/g at 20 °C.

### Fluorescence measurements.

Steady-state fluorescence measurements were performed using a SPEX Fluoromax instrument (Instruments S.A., Inc., Edina, New Jersey, United States). Excitation and emission bandpasses were 5 nm and the cuvette holder was temperature controlled to 37 ±0.1 °C (Thermo-Neslab, Portsmouth, New Hampshire, United States). To eliminate contributions from amino acid residues other than W and to minimize absorbance by acrylamide, the excitation wavelength was 295 nm. Emission spectra were recorded from 310 to 420 nm using GLTP concentrations (1 μM) with an optical density of less than 0.1 at 295 nm to avoid the inner filter effect. Measurements were performed under constant stirring by addition of small defined aliquots of GSL dissolved in ethanol (1 mM) to a fixed volume of protein. Rapid equilibration of the fluorescence emission signal was observed (2–3 min) with no additional change observed by additional incubation up to 20 min.

### Crystallization and x-ray data collection.

Crystals of glycolipid-GLTP complexes were obtained by cocrystallization of protein with glycolipid using the hanging drop technique. Protein samples (10–15 mg/ml) were mixed with glycolipid dispersion (0.4 mM in 20%–40% EtOH), and then with well solution at ratio 1:1:1 resulting in a pH near 6.0. The well solution contained 15%–20% (weight/volume) PEG 3350 (or PEG 8000), 50 mM KH_2_PO_4_, pH 4.5. Single crystals appeared in 2 wk to 3 mo, depending on glycolipid type. All GSL-GLTP crystals were isomorphous and belonged to the space group *C2*, with unit cell parameters indicated in Table 1. GLTP complex with *n*-hexyl-β-d-glycoside, which also crystallized in the *C2* space group, is characterized by different unit cell (Table 1) and two molecules in the asymmetric unit (AU), in contrast with GSL-GLTP crystals, which all have one molecule in AU. Apo-GLTP crystals belonged to the *P2*_1_ space group [16,49]. Crystals were transferred into well solution including 20% glycerol as cryoprotectant, next mounted in a fiber loop and flash-frozen in a cold nitrogen stream for x-ray data collection. All data sets were collected on a Rigaku RU-H3R X-ray generator (Rigaku Americas, The Woodlands, Texas, United States) equipped with a RAXIS-HTC detector. Data collection statistics are presented in Table 1.

### Structure determination and refinement.

Because the GSL-GLTP crystals reported in this paper are isomorphous to crystals of the 18:1 LacCer-GLTP complex [16], the coordinate set of this crystal structure (with ligand omitted) was used as an initial model for the 24:1 GalCer-GLTP complex (Table 1, column 1). The 24:1 GalCer was added with the help of the difference electron density map. The model was refined in REFMAC [50] at 1.85 Å resolution to a final *R*-factor/*R*-free of 0.180/0.230 (Table 1). The automatic procedure ARP/wARP was used to add solvent molecules to a final model [51]. The simulated annealed omit 2*F*_o_-*F*_c_ map for the ligand region is shown in Figure 1D. After appropriate deletions, the model was applied as the initial one for all other GSL-GLTP complexes, which were refined at an appropriate resolution to *R* factors shown in Table 1. The structure of GLTP complex with *n*-hexyl-β-d-glucoside was determined by the molecular replacement (MR) method via the AMoRe program [52], and then refined in REFMAC. The model used in the MR search was native apo-GLTP.

### Evaluating general protein-protein interface propensity.

The protocols for the ODA computations [29] are as follows: a mesh of points, located 3 Å above the protein surface, was generated with an average distance of 5 Å between them. For series of spheres centered at each point and characterized by a variable radius, changing from 5 Å to 20 Å, the product of the atomic solvent accessible areas by the atomic ODA densities [29] was calculated. The optimal radius was then selected and the best desolvation value was assigned to the residues. The method was previously validated on 66 nonhomologous protein pairs involved in nonobligate protein-protein complexes of known structure.

### Estimating the binding energy for GLTP complexes.

The binding energies between two monomers with and without ligands were calculated using the method described in [53]. The binding energy values were calculated using an empirical formula consisting of three main contributions: (1) the electrostatic free energy difference evaluated using the boundary element solution of the Poisson equation [54], (2) the surface energy difference, and (3) the entropic loss estimate. The parameters used in calculations, including the constant C = 7 kcal/mol, the surface energy density 0.03 kcal/Å^2^, and the interface dielectric constant of 8, were optimized previously on a diverse set of protein-peptide and protein-protein complexes. The expected accuracy was shown to amount to 2.5 kcal/mol.

### Calculating the contact surface areas.

The contact surface area was calculated using the fast implementation of the Shrake and Rupley algorithm implemented in the ICM program [55], using the probe radius of 1.4 Å and the van der Waals radii as follows: C aliphatic, 1.95 Å; C aromatic, 1.8 Å; N, 1.7 Å; OH, 1.6 Å; O, 1.4 Å; SH, 2.0 Å; and S, 1.8 Å. The contact surface area was calculated as Area(A) + Area(B) − Area(AB).

## Supporting Information

Figure S1 Structure of Apo-GLTP (A) Structure of the apo-GLTP [16], with α helices shown in a gold cylinder representation, whereas 3_10_ helices are shown in silver ribbon representation. Extraneous hydrocarbon is shown in a silver space-filling representation. (B) The portal of the apo-GLTP form with no residues removed from the GLTP electrostatic representation. The extraneous hydrocarbon end seen from the portal is shown in a silver space-filling representation.
(6.0 MB TIF)

Figure S2 Structure of the 18:1 LacCer-GLTP Complex (A) Chemical formula of 18:1 lactosylceramide. (B) Crystal structure of the 18:1 LacCer-GLTP complex [16]. The GLTP is shown in a gold ribbon representation, whereas the carbon atoms of the LacCers are shown in a lavender-colored space-filling representation. (C) Stick representation of the sphingosine-in conformation of the 18:1 LacCer (carbon atoms colored in lavender) in the complex.
(2.2 MB TIF)

Figure S3 Protein-Protein and Protein-Membrane Interaction Propensities Calculated by the ODA Approach for Three Different GLTP Complexes for Views Emphasizing Crystal-Related Dimerization (A) Complex of GLTP with *n*-hexyl-β-d-glucoside. (B) Sphingosine-in GLTP complex with 18:1 LacCer. (C) Sphingosine-out GLTP complex with 24:1 GalCer. Structures are colored by the absolute magnitude of the ODA signal from the strongest in red, through medium in orange, weak in yellow, to the weakest in gray.
(4.4 MB TIF)

Figure S4 Crystal-Related Cross Dimerization of GLTP Complexed with Different GSL Ligands (A) Crystal-related cross dimer in the structure of the 8:0 LacCer-GLTP complex. The GLTPs are shown in a gold ribbon representation, whereas the carbon atoms of the LacCers are shown in a cyan space-filling representation. (B) Crystal-related cross dimer in the structure of the 18:2 GalCer-GLTP complex. The GLTPs are shown in a gold ribbon representation, whereas the carbon atoms of the GalCers are shown in a lemon-colored space-filling representation.
(5.3 MB TIF)

Figure S5 Structure of the 18:2 GalCer-GLTP Complex (A) Chemical formula of 18:2 galactosylceramide. (B) The crystal structure of the 18:2 GalCer-GLTP complex. The GLTP is shown in a gold cylinder representation, whereas the carbon atoms of the GalCers are shown in a lemon-colored space-filling representation. (C) Superposition of stick representations of the 18:2 GalCer (carbon atoms colored in lemon) and 18:1 LacCer (carbon atoms colored in lavender) derived from their sphingosine-out and sphingosine-in GLTP complexes, respectively. Arrows point out the positions of *cis* double bonds in acyl chains.
(2.1 MB TIF)

### Accession Numbers

The coordinates and diffraction amplitudes from this study have been deposited in the Protein Data Bank (http://www.rcsb.org/pdb/) with the following accession numbers: 24:1 GalCer-GLTP complex (2EUK); 8:0 LacCer-GLTP complex (2EUM); 12:0 LacCer-GLTP complex (2EVD); 18:2 GalCer-GLTP complex (2EVL); *n*-hexyl-β-d-glucoside-GLTP complex (2EVS); and D48V mutant apo-GLTP (2EVT). The previously deposited coordinates for apo-GLTP and 18:1 LacCer-GLTP have accession numbers 1SWX and 1SX6, respectively[16]. The GenBank (http://www.ncbi.nlm.nih.gov/Genbank/) accession numbers for open reading frame encoding human GLTP are AF209704, AY372530, AY372531, and AY372532.

## Abbreviations

GalCer: galactosylceramide
GLTP: glycolipid transfer protein
GSL: glycosphingolipid
LacCer: lactosylceramide
ODA: optimal docking area
